# Determination of Thyroid Volume by Ultrasonography among Schoolchildren in Philippines

**DOI:** 10.1155/2012/387971

**Published:** 2012-05-22

**Authors:** Bu Kyung Kim, Young Sik Choi, Chul Ho Oak, Yo-Han Park, Jae Hyun Kim, Dae Jin Park, Cindy Mora, Donald Wilson, Eun-Kee Park

**Affiliations:** ^1^Department of Internal Medicine, Kosin University College of Medicine, Busan 602-702, Republic of Korea; ^2^Kosin university Gospel Hospital, Amnam-dong, Seo-gu, Busan 602-702, Republic of Korea; ^3^Department of Pharmacology, Kosin University College of Medicine, Busan 602-702, Republic of Korea; ^4^Department of Occupational Toxicology, Institute of Industrial Ecological Sciences, University of Occupational and Environmental Health, Kitakyushu, Japan; ^5^Department of Medical Humanities and Social Medicine, Kosin University College of Medicine, Busan 602-702, Republic of Korea

## Abstract

*Objective*. Iodine deficiency is defined by the goiter and the urinary iodine concentration. However, a lack of local thyroid volume reference data resulted in the vague definition of goiter, especially in school-aged children. The aim of this paper was to determine the thyroid volumes by ultrasonography in schoolchildren aged 6 to 12 years living in Cagayan areas in Philippine. *Methods*. Cross-sectional thyroid ultrasonographic data of 158 schoolchildren aged 6–12 years from Tuguegarao and Lagum in Cagayan valley, Philippine were used. Thyroid volumes were compared based on logistic issue and urban and rural area and compared with other previously reported data. *Results*. The mean values of thyroid volume in Tuguerago and Lagum were 2.99 ± 1.34 mL and 2.42 ± 0.92 mL. The thyroid size was significantly in association with age (*P* < 0.00), weight (*P* < 0.00), height (*P* < 0.00), and BSA (*P* < 0.00) by Pearson's correlation. The median thyroid volumes of schoolchildren investigated in this study were generally low compared to international reference data by age group but not by BSA. *Conclusions*. We propose for the first time local reference ultrasound values for thyroid volumes in 6–12 aged schoolchildren that should be used for monitoring iodine deficiency disorders.

## 1. Introduction

Thyroid diseases are a common endocrine disease among children and adolescents. Iodine deficiency disorders (IDDs) are a global problem, affecting the people of 130 of 191 member countries of the World Health Organization. It is one of the most common preventable causes of mental retardation globally [5, 6]. For several decades, thyroid palpation was the standard method for determining thyroid size. Palpation method has been used in field survey of endemic goiter. The World Health Organization (WHO) and the International Council for the Control of IDDs (ICCIDD) defined the endemic goiter as the case when more than 5% of the school-age population have goiter [7, 8]. Although palpation is simply performed with minimal cost, it is difficult to determine thyroid size precisely. Particularly, it has been inadequate for distinguishing mild thyroid enlargement from normal according to decreasing of iodine deficiency worldwide [9].

 Thyroid ultrasonographty is a reliable procedure for the assessment of thyroid size. Ultrasonographic examination of the thyroid provides precise information on thyroid volume and structure and now is considered the most reliable method of determining thyroid volume [10–12]. However, to use ultrasonographic method for determination of thyroid volume reliable reference data should exist. Unfortunately an appropriate standard data is still lacking. WHO/ICCIDD recommended European result by Delange et al. as an international reference for thyroid volume in children [13]. However there were different reference values available [11, 14–17]. These discrepancies concluded the need to establish specific local values in any population with adequate iodine intake.

 Through universal salt iodization (USI), amazing progress has been made in reducing iodine deficiency in Asia [18]. USI may be the most successful public health effort of the past two decades. In the Philippines, Republic Act No. 8172 promoting salt iodization nationwide, otherwise known as the ASIN Law, was passed in 1995. This law requires iodization of all salt for animal and human [19]. Goiter surveys were conducted by Philippines National Nutrition Surveys (NNSs) in 1987 only targeting adults and found an overall goiter prevalence of 12%, for the six regions (I, II, III, IV, VI, and X). The overall prevalence for these six regions was 12% [20]. In the 1993 NNS, The goiter rate increased to 6.7% [21]. In both NNS, goiter was determined by palpation. In the succeeding surveys of 1998, 2003, 2008 goiter prevalence was not determined. So there was no reference value measured by ultrasonography for school-aged children in Philippines.

 In this paper, we examined the thyroid volume of 6–12-year-old children in Cagayan in Philippines by ultrasonography and compared them with other findings [1–4]. Cagayan belongs to Region II; in the 1987 NNS goiter prevalence of this region was Oriental Mindoro (56%), Marinduque (36%), Bantagas (17%), and Cavite (15%) [20]. The aim of this paper was to determine the thyroid volumes by ultrasonography in schoolchildren aged 6 to 12 years living in Cagayan areas in Philippine, which are known as the areas of iodine deficiency.

## 2. Subjects and Methods

### 2.1. Study Population

 This study was conducted in Cagayan valley, Phillipines between June and July 2011. The subjects were 158 schoolchildren aged 6–12 years old. Among them were 49 schoolchildren (27 boys and 22 girls) living in Tuguegarao which is one of the biggest cities in the Cagayan valley and 109 (52 boys and 56 girls) living in Lagum which is the rural area. Body weight (kg) and height (cm) in all participants were measured to determine body surface area (BSA). The BSA was calculated by the following formula [22]:

(1) $$\text{BSA}=\text{Weight}\;{\left(\text{Kg}\right)}^{0.425}\times \text{Height}\;{\left(\text{cm}\right)}^{0.725}\times 71.84\times 10^{-4}.$$

This study was approved by Cagayan State University in Tuguegarao City, Cagayan, Philippines and Kosin University College of Medicine, Busan, Republic of Korea. Informed consent was obtained from the parents of the children.

### 2.2. Thyroid Volume

 Thyroid volume was estimated by using ultrasound (LOGIQ BOOK XP, GE healthcare, Seoul, Korea) portable instrument, with a 10 MHz linear transducer. The examination was performed by two experienced internal medicine specialists. The subjects were supine position with the hyperextension of neck for examination. The thyroid volume of each lobe was calculated using the formula [23]: width (cm) × length (cm) × depth (cm) × 0.479 for each lobe. The thyroid volume was the sum of the volume of both lobes. Isthmus volume was not taken into account.

### 2.3. Urinary Iodine Concentration

Urinary iodine (UI) concentrations were performed from only the 80 children in Lagum. The spot urine samples (4.0 to 11.5 mL) were collected in wide-mouthed screw capped plastic bottle. The samples were immediately refrigerated after being collected until analysis. UI was measured with an iodine selective electrode method by Orion 4-Star Plus pH/ISE Portable Meters (Thermo Fisher Scientific, Beverly, MA, USA). Iodine concentration was expressed by *μ*g/L, the iodine deficiency grade was defined according to the WHO's median UI level criteria [6]. 

### 2.4. Statistical Analysis

For each subject, the observed measurements were carried out by the two physicians who were averaged to determine definitive values of thyroid volume. The data obtained from Tuguegarao and Lagum regions was compared by student's *t*-test. Relationships among weight, height, BSA, and thyroid volume were analyzed by Pearson's correlation coefficient. Thyroid volume was represented by the value of minimum, maximum, median, and mean ± standard deviation. Multivariate linear regression test was performed in order to determine the factors that affect thyroid volume. We compared out thyroid volume results with previous studies and the recommended normal values established by WHO/ICCIDD. Statistical analysis was performed using the Statistical Package for Social Sciences (SPSS, version 17.0, Chicago, IL, USA). 

## 3. Results

### 3.1. Regional Comparisons

**Figure 1:**
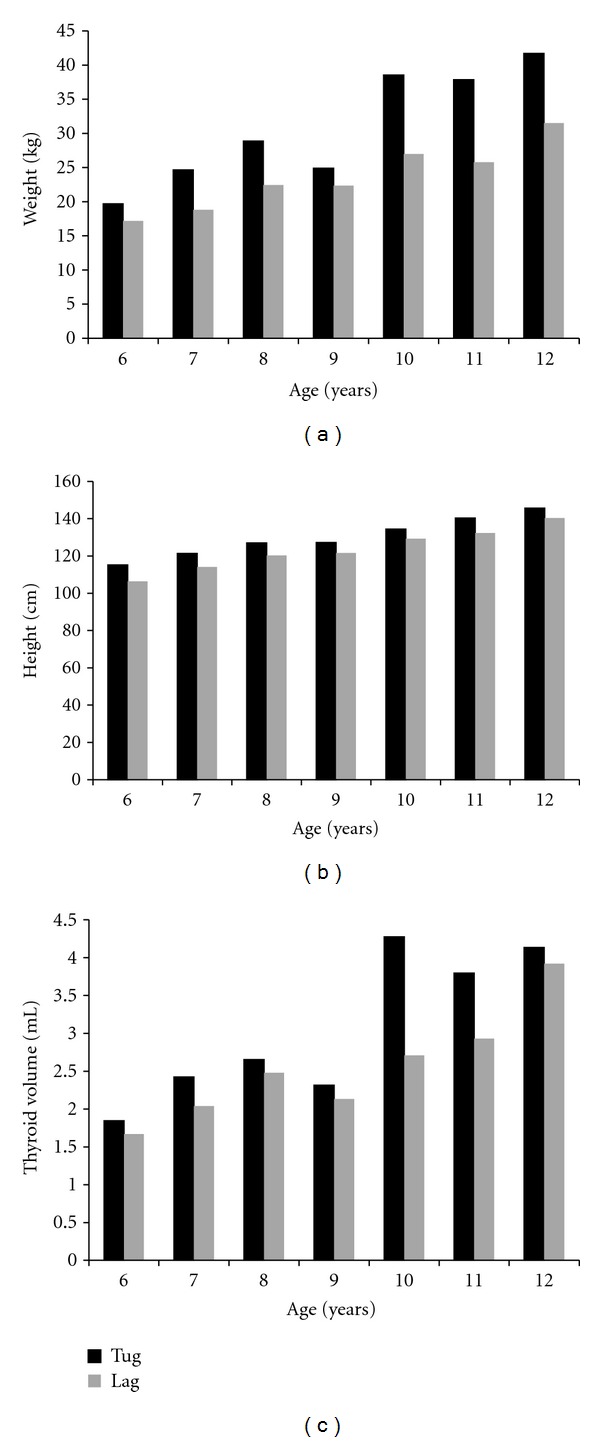
In all age subgroup (six to twelve), (a) weight, (b) height, and (c) thyroid size were smaller in Lagum than Tuguegarao.

 The baseline characteristics were presented based on investigated areas (Table 1). The mean (SD) age was same between the children in Tuguegarao and Lagum areas. The mean weight was 29.7 ± 9.4 kg in Tuguegarao and was 22.7 ± 5.4 kg in Lagum. The mean height, BSA, and thyroid size were 128.04 ± 10.72, 1.02 ± 0.20, and 2.99 ± 1.34 in Tuguegarao, respectively, and 121.43 ± 11.97, 0.87 ± 0.14, and 2.42 ± 0.92 in Lagum, respectively. In all age subgroup (six to twelve), weight, height, and thyroid size were smaller in children of Lagum than Tuguegarao (Figure 1).

### 3.2. Thyroid Volume

**Figure 2:**
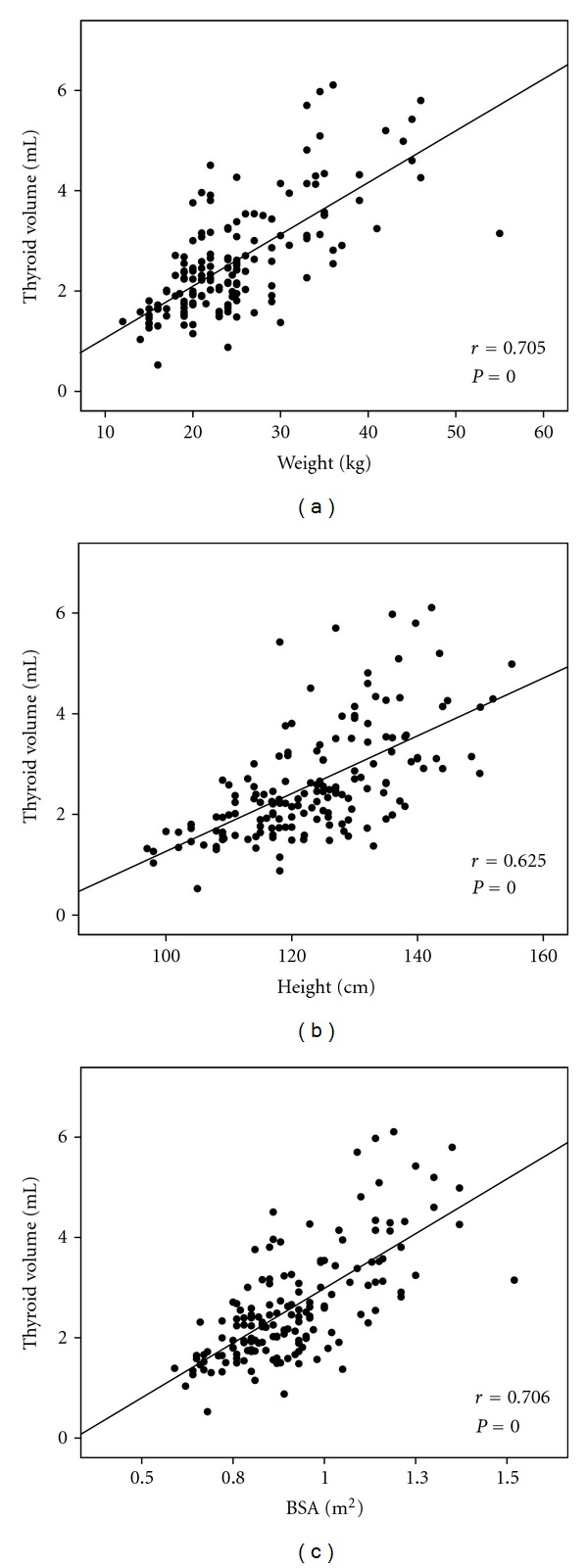
Scatter plots show the significant relationship of thyroid volume with (a) weight, (b) height, and (c) BSA.

The thyroid size was significantly in association with age, weight, height, and BSA by Pearson's correlation which is presented in Figure 2 (*P* values). However, after being adjusted with age, weight, and height, only age (*P* = 0.022) and weight (*P* = 0.000) had significant effects on the thyroid size by the multiple linear regression analysis (Table 2).  Table 3 shows the values of this report for the lowest, largest, median, and mean thyroid volume measured by ultrasonography in Philippines according to age.  Table 4 shows the values for the lowest, largest, median, and mean thyroid volume measured by ultrasonography in Philippines according to BSA (Table 4).

### 3.3. Comparison with International References

**Figure 3:**
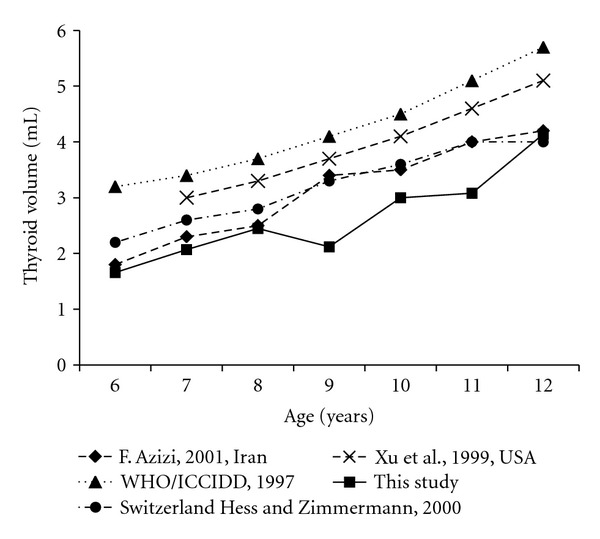
Median (P50) thyroid volume according to age in 6–12-year-old Filipino compared with international reference data.

**Figure 4:**
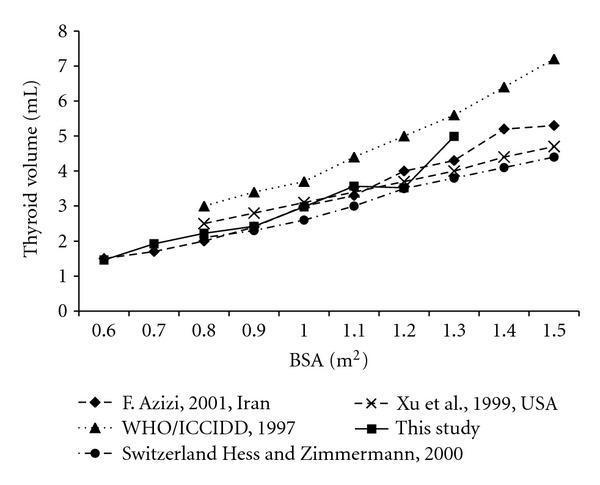
Median (P50) thyroid volume according to BSA in 6–12-year-old Filipino compared with international reference data.

The comparison of this study to the data from other countries is presented in Figures 3 and 4.  Figure 3 shows the median thyroid volumes of Filipino schoolchildren were generally lower than those observed in other countries (Europe, Iran, and United States of America) by age group. However it was reported that the median thyroid volumes according to BSA of Filipino schoolchildren were similar to those of other data except the reference of WHO/ICCIDD (Figure 4).

### 3.4. Urine Iodine Concentration

 The median urinary iodine concentration of 80 samples in Lagum was 279 *μ*g/L (ranged between 20.8 and 874 *μ*g/L), and 12.4% of children had insufficient of urinary iodine concentration (<100 *μ*g/L).

## 4. Discussion

 In the present study, we represented the thyroid volume measured by ultrasonography in 6–12-year-old children from Cagayan valley in Philippines. BSA, weight, and height were the best predictor of thyroid volume. Foo et al. [11] proposed BSA-specific reference could be different from age-based reference. The authors conceded that BSA specific upper limit might be a larger reference. In this study thyroid volume was significantly in correlation with weight, height, and BSA. We presented the data according to age and also BSA.

In this study, we compared the thyroid volume, weight, height, and BSA of schooled aged children in two areas urban and rural areas. The mean thyroid volume was larger in urban than rural. It is because the weight and height of children in urban areas were larger than in rural ones. So we could guess the children in urban areas have better nutritional status than those in rural ones. Unfortunately, urine samples were taken from only children in rural ones. In this study, 12.4% of children had insufficient urinary iodine concentration. If we assume that nutritional status of children in urban areas is better than that in rural, we can expect that the size of iodine insufficiency of total subjects is smaller than 12.4%. Actually iodine deficiency is overcoming in the Philippines, and the median urinary iodine concentration of 6–12 years children was 132 *μ*g/L according to 2008 NNS [24].

The thyroid volumes of this study were smaller than the reference data of WHO/ICCIDD in 1997. The reference was based on Europe which is an iodine deficiency area. Surprisingly, Europe is still the highest iodine-deficient area in the world [25]. Interestingly, to compare other reports from iodine-sufficient area, the thyroid volumes of this study were smaller than other studies by age, but similar to other studies by BSA. We could guess it was because weights and heights of children in Philippines were smaller than other countries. But we could not compare specifically because there was no data of weight and height by age in other studies.

Of course thyroid volume could not be explained wholly by weight and height. In this study, the value of *R* square was 0.521 by multivariate linear regression with age, weight, and height. So 48% of thyroid volume was determined by other factors like as ethnicity. Therefore previous reports suggest the need for population-specific reference. So far, several countries have been presenting their own reference data [26–30]. Particularly Sweden has their own data of BSA-specific references by age [30]. We expect Philippines would have such reference through future studies.

 This study has two limitations. The first, as mentioned earlier, urine samples were taken from only children in rural. The sample size is not enough to conclude a final decision. So we couldnot present the values of 97 percentile. The reference value of thyroid volume is necessary to determine goiter prevalence. So it is important to which value will be considered as normal. Therefore it is critical limitation that there was no data of 97 percentile. Instead we present largest value by age and BSA. It will be reference of future studies.

Despite of these limitations this study is worth. This is the first reference of thyroid volume measured by ultrasonography in Philippines. So it will be valuable regional reference for next studies about iodine deficiency and thyroid would perform in Philippines. 

## Figures and Tables

**Table 1:** The baseline characteristics in investigated areas.

	Total	Tuguegarao	Lagum	*P* value
	*n* = 158	*n* = 49	*n* = 109	
Age (year)	8.6 ± 1.9	8.6 ± 1.9	8.6 ± 1.9	0.939
Weight (kg)	24.80 ± 7.59	29.67 ± 9.40	22.66 ± 5.44	0.000
Height (cm)	123.45 ± 11.97	128.04 ± 10.72	121.43 ± 11.97	0.001
BSA (m^2^)	0.92 ± 0.18	1.02 ± 0.20	0.87 ± 0.14	0.000
Thyroid size (mL)	2.60 ± 1.10	2.99 ± 1.34	2.42 ± 0.92	0.002

**Table 2:** Associations of thyroid volume with age, weight, and height by multiple linear regression analysis.

	*β* ± SE	*R* ^2^	Coefficient	*P* value
Age	0.128 ± 0.055	0.521	−0.236	0.022
Weight	0.085 ± 0.014			0.000
Height	−0.003 ± 0.012			0.795

SE: standard errors.

**Table 3:** The lowest, largest, median, and mean thyroid volume measured by ultrasonography according to age in investigated areas.

Age (year)	Subject (*n*)	Minimum (mL)	Maximum (mL)	Median (mL)	Mean (mL) ± SD
6	29	0.53	3.16	1.66	1.72 ± 0.57
7	26	1.15	3.80	2.07	2.18 ± 0.66
8	23	1.46	4.51	2.45	2.52 ± 0.77
9	22	1.37	3.75	2.12	2.18 ± 0.62
10	27	1.67	5.79	3.00	3.29 ± 0.84
11	19	1.90	6.1	3.08	3.20 ± 0.99
12	12	2.16	5.98	4.14	3.97 ± 1.12

**Table 4:** The lowest, largest, median, and mean thyroid volume measured by ultrasonography according to BSA.

BSA (m^2^)	Subject (*n*)	Minimum (mL)	Maximum (mL)	Median (mL)	Mean (mL) ± SD
0.6	15	0.53	2.62	1.46	1.52 ± 0.49
0.7	24	1.32	3.00	1.92	2.00 ± 0.45
0.8	45	0.88	4.51	2.22	2.33 ± 0.78
0.9	31	1.48	4.26	2.42	2.43 ± 0.65
1.0	14	1.37	5.7	2.98	3.03 ± 1.13
1.1	17	2.29	6.11	3.57	3.89 ± 1.14
1.2	6	2.81	5.42	3.52	3.75 ± 0.99
1.3	6	4.26	5.80	4.99	4.97 ± 0.59
